# A New Method for *in Situ* Measurement of Bt-Maize Pollen Deposition on Host-Plant Leaves

**DOI:** 10.3390/insects2010012

**Published:** 2011-02-21

**Authors:** Frieder Hofmann, Mathias Otto, Ulrike Kuhn, Steffi Ober, Ulrich Schlechtriemen, Rudolph Vögel

**Affiliations:** 1 TIEM Integrated Environmental Monitoring GbR, Nörten-Hardenberg/Bremen, Germany; E-Mail: tiem@arcor.de (U.S.); 2 Ökologiebüro, Bremen, Germany; E-Mail: f.hofmann@oekologiebuero.de (F.H.); 3 Federal Agency for Nature Conservation, Department of Biosafety, Bonn, Germany; E-Mail: ottom@bfn.de; 4 Büro Kuhn, Bremen, Germany; E-Mail: kuhn-bremen@t-online.de; 5 IFÖN, Berlin, Germany; E-Mail: steffi.ober@nabu.de; 6 Sachverständigenbüro, Nörten-Hardenberg, Germany; E-Mail: uschlechtriemen@arcor.de (U.S.); 7 Environmental Agency Brandenburg (LUA), Eberswalde, Germany; E-Mail: rudi.voegel@lua.brandenburg.de

**Keywords:** GMO, GMP, Bt maize, maize, pollen, deposition, exposure, non-target organisms, Lepidoptera

## Abstract

Maize is wind pollinated and produces huge amounts of pollen. In consequence, the Cry toxins expressed in the pollen of Bt maize will be dispersed by wind in the surrounding vegetation leading to exposure of non-target organisms (NTO). NTO like lepidopteran larvae may be affected by the uptake of Bt-pollen deposited on their host plants. Although some information is available to estimate pollen deposition on host plants, recorded data are based on indirect measurements such as shaking or washing off pollen, or removing pollen with adhesive tapes. These methods often lack precision and they do not include the necessary information such as the spatial and temporal variation of pollen deposition on the leaves. Here, we present a new method for recording *in situ* the amount and the distribution of Bt-maize pollen deposited on host plant leaves. The method is based on the use of a mobile digital microscope (Dino-Lite Pro, including DinoCapture software), which can be used in combination with a notebook in the field. The method was evaluated during experiments in 2008 to 2010. Maize pollen could be correctly identified and pollen deposition as well as the spatial heterogeneity of maize pollen deposition was recorded on maize and different lepidopteran host plants (*Centaurea scabiosa*, *Chenopodium album*, *Rumex spp.*, *Succina pratensis* and *Urtica dioica*) growing adjacent to maize fields.

## 1. Introduction

Insect resistance is one of the main traits introduced in genetically modified plants (GMP) worldwide and Bt plants account for approximately 15% or 22 million hectares of the total area cultivated with GMP [1]. Bt plants produce one or several kinds of Bt proteins from the soil bacterium *Bacillus thuringiensis* that are toxic to insect pests such as lepidopteran pests of maize or cotton (e.g., *Ostrinia nubilalis* or *Helicoverpa zea*) or coleopteran pests of maize (e.g., *Diabrotica virgifera*). Because the Bt proteins are usually expressed in all plant parts, pollen containing the protein will be dispersed by the wind into ecosystems adjacent to the field. As this pollen is deposited on the vegetation it may cause exposure and possible toxic effects to non-target herbivores. Therefore, the assessment of possible harmful effects of Bt proteins on non-target organisms (NTO) has a high priority in the environmental risk assessment (ERA). Such an assessment is mandatory as the cultivation of GMP is regulated.

For ERA, risk is defined in terms of a combination of hazard x exposure [2,3]. Exposure assessment is therefore an important and basic step in the risk assessment of GMP. In the case of Bt maize, direct effects outside the field will mainly depend on the uptake of maize pollen by NTO and their sensitivity to the Bt proteins. To date, various lepidopteran active Bt proteins such as Cry1Ab, Cry1A.105, Cry1F, or VIP3A have been incorporated in Bt maize and the deposition of pollen from these fields in nearby habitats has been identified as a hazard for non-target Lepidoptera [4].

Information to estimate exposure can be collected in various ways and with different methods. In fact, pollen monitoring has been carried out for more than a century and several methods have been reported in literature, mainly adapted to the purpose of the particular research [5]. Basically, there are three main parameters to be distinguished: pollen concentration, flow and deposition on surfaces. Whereas pollen concentration and flow in the air are physically defined parameters determined only by the amount of pollen present in the air, deposition depends on the acceptor's surface too, e.g., the condition of the leaf surface. Consequently, even if pollen concentration and flow in the air are constant, pollen deposition on the leaves may vary between different host plants depending on the respective leaf conditions. Volumetric traps are active samplers with an air pump or motor where the measurement can be assigned to a defined air volume aiming to measure pollen concentration in the air. Commonly used in Europe is the Hirst-type [6], known under the brand names “Burkard” and “Lanzonii”. In the U.S., the Rotorod, a motor driven rotating impactor [7], is used. Various methods have been applied to estimate maize pollen deposition but systematic direct measurements of pollen deposition on host plants are rare because of methodical difficulties. Gravimetric methods like slides or petri dishes coated with adhesive belong to the oldest methods and are widely used. The method is simple, but due to physical reasons it is suitable only for semi-quantitative measurements [5,8]. A more advanced device is the Durham trap [9,10], where the slide with the coated surface is protected from direct influences like rain. A passive sampling system that could be standardized successfully [11] is the technical pollen sampler Sigma-2/Pollen-Mass-Filter (PMF), which measures deposition and flow [12,13].

Apart from pollen traps, pollen deposition has been derived from plant surfaces in few cases but rather in an indirect way. Methods to do so included washing off the pollen [14], using adhesive strips or cement for so-called ‘leaf prints’ [15–17] or removing plant leaves or part of them from the field into the lab to examine them microscopically [14]. Because pollen and/or leaves are removed by these methods, processes like pollen accumulation or dislocation cannot be observed directly, and in consequenc,e temporal and spatial variation can only be estimated indirectly.

Here we present a new method which allows *in situ* observation of pollen deposition on host plants by using a portable digital microscope. The method involves minimal handling of the plant and allows in contrast to other methods, direct measurement of the spatial variation of pollen deposition on the plant surface as well as repeated measurements of the same leaf.

Although the method can be used for various purposes, it has been developed in the context of GMO risk assessment. Information on the spatial distribution of pollen on host plants is of importance for the assessment of exposure of NTO to Bt pollen as some butterfly larvae may preferably feed on certain leaf parts and the information on the distribution of pollen is necessary to calculate the maximum amount of pollen larvae may be exposed to. As technical samplers only measure mean pollen densities such data has so far rarely been collected.

In this publication, we focus mainly on the method itself and give some examples of the spatial distribution and quantitative data recorded for different host plants. Due to the importance of the maximum amount and the variability of pollen deposition on plant leaves for the risk assessment, special focus has been given to these aspects.

## 2. Experimental Section

**Figure 1:**
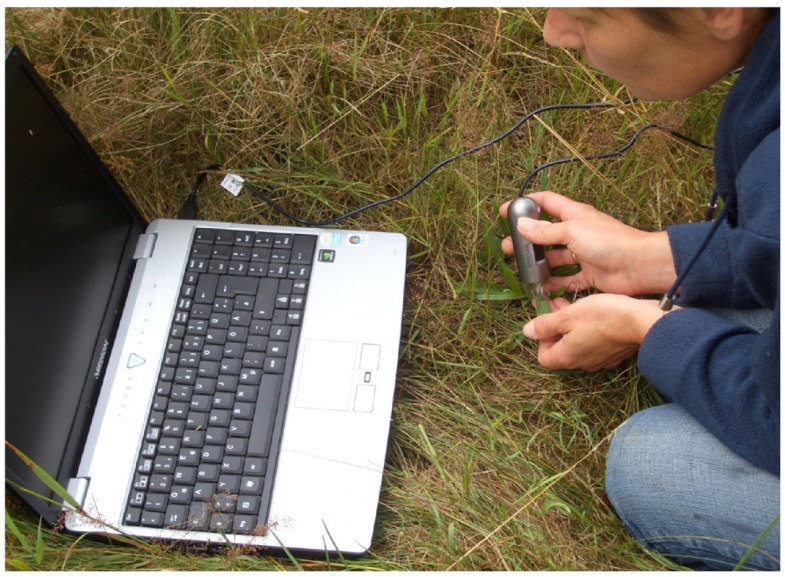
Use of the digital microscope for *in situ* measurement of maize pollen in the field. The microscope is powered via the USB hub, the labtop monitor serves to verify the images taken.

Digital imaging, including microscopy, has made tremendous advances during the last decade and digital microscopes have been used in medicine and in industrial inspection and quality control for many years. In our experiments, we used a digital microscope with a resolution of 1.3 megapixels (Dino-Lite Pro AM413MT; AnMo Electronics Corporation). The portable microscope is easy to handle with a total length of 10 cm and can be powered via the USB hub of a notebook. Figure 1 depicts the use of the USB powered microscope in the field. The microscope model used has a fixed focus, build in LED-lights and a magnification which can be adjusted from 10–70× and 200×. A measurement and calibration software (DinoCapture) is included by the manufacturer.

**Figure 2:**
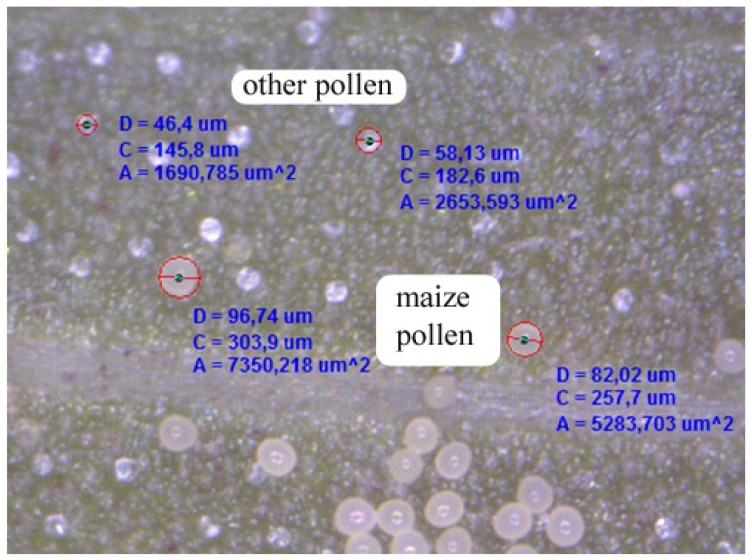
Image of upper leaf side of *Centaurea scabiosa* taken *in situ* with Dino-Lite microscope (200×). Maize pollen can be distinguished from other pollen species by its relatively large size between 80–120 μm, color, shape and structure of the exine. Measurements are given by Dino Capture software.

The size of the sampling spot varies with the magnification of the microscope between approximately 20 mm^2^ (50×) and 5 mm^2^ (200×) and was calibrated before measurement. A magnification of 200× is preferable for most pollen counts as maize pollen can be more easily identified with this magnification (Figure 2). Pollen analysis was done visually. The use of image analysis to quantify pollen seems possible but is restricted to occurrences of lower pollen densities when pollen does not overlap. Such overlap will interfere with automatic image analysis and result in additional effort for quality control, including a visual re-analysis of images. Our first aim was to test whether maize pollen can be identified and distinguished from other pollen-types and whether the method is suitable for *in situ* measurements of different plant and leaf types. Criteria for the selection of plant species were the availability in and close to the maize fields, leaf shape, presence/absence of leaf hairs, and the plant's potential role as host plant for lepidopteran larvae. The plant species selected, apart from maize, were stinging nettle (*Urtica dioica*), goosefoot (*Chenopodium album*), two dock species (*Rumex crispus* and *R. obtusifolius*), devil's scabious (*Succisa pratensis*), and knappweed (*Centaurea scabiosa*).

Due to the great variation of maize pollen deposition on the leaves, our second aim was to compare different sample designs to optimize the assessment of the variation of the pollen deposition using maize as a model plant. Designs compared were: (i) Random raster: The leaf was divided into a raster of 19 lateral sections of 2.5 cm width and five longitudinal sections of 1.75 cm. 35 raster points were randomly assigned. (ii) Full lateral transect: One transect in the middle of the leaf was analyzed by 38 consecutive images. (iii) Repeated lateral transects: five transects were distributed over the leaf and analyzed by five measuring points each (mid rip, two spots mid leaf; leaf edges). (iv) Clusters each with 5 measuring points. Two clusters were taken in areas with high and low pollen deposition, respectively. Data from designs i-iv were pooled to a total data set. Descriptive statistics on the data distribution such as the mean, minimum, maximum as well as the the 95% and 5% quantiles were calculated for each of the designs and for the pooled data on basis of the log-normal distribution. Zero values were assigned to two-thirds of the detection limit (14 pollen/cm^2^). Consecutively, a structured design was derived combining three transects with five measuring points each and four clusters with three sample points each (two of areas with high and two of areas with low pollen densities) using the statistical program R.

Third, the method was tested *in situ* taking images from different plant species in different locations in the state of Brandenburg, Germany, during maize anthesis in the years 2008 to 2010. All plants were located within close proximity (up to 20 m) to the maize field margin and sampled with the derived structured sampling design. Here we present some results as an example, focusing on the ease of application of the method.

## 3. Results and Discussion

**Figure 3:**
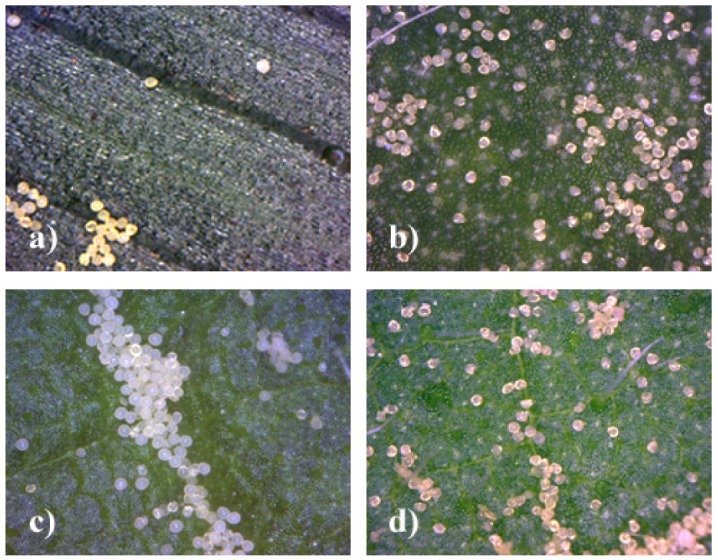
Images taken *in situ* with Dino-Lite microscope (200×), upper leaf side. (**a**) Zea mays 540 pollen/cm^2^; (**b**) *Chenopodium album*, 2800 pollen/cm^2^; (**c**) *Rumex* spp., 3600 pollen/cm^2^; (**d**) *Urtica dioica*, 2400 pollen/cm^2^.

**Figure 4:**
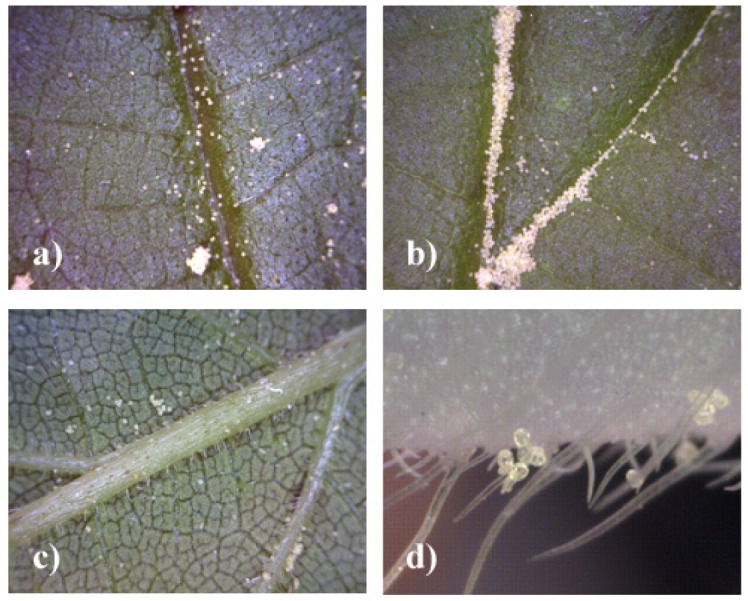
Images taken *in situ* with Dino-Lite microscope. (**a**, **b**) Examples of pollen accumulation on *Urtica dioica* on upper leaf side (50×). (**c**) Leaf underside of *Urtica dioica* (50×); (**d**) Sideways view on leaf of *Succisa scabiosa* (200×).

Using the portable digital microscope it is possible to obtain direct, *in situ* measurements of maize pollen deposition on plant leaves. Examples of images are given in Figures 2–4. Maize pollen could be accurately identified by visual analysis under 200× magnification according to its relatively large size (usually 80–120 μm) and characteristic shape and color (Figure 2). We found that the method can be applied well *in situ* for identifying maize pollen. Although the method may be applied to other pollen species, it will need to be checked and adapted, especially for smaller pollen. The method involves minimal handling of plant leaves and is not destructive. As a result, the maize pollen deposition on the leaves can be observed repeatedly, which offers the opportunity to obtain data on the temporal and spatial variation of pollen deposition on the leaves during anthesis in a representative way. By the method it is further possible to obtain information on pollen densities from both the leaf upper side and leaf underside (Figure 4). In addition, sideway images of leaves can be taken to visualize, for example, the role of leaf structures such as trichomes (e.g., *S. scabiosa*, Figure 4d). For *in situ* measurements in the field, two people are recommended, one working with the microscope on the leaf and one with the notebook. As the microscope is powered by the laptop, the operating time of the notebook without an external power supply is limited.

The images taken clearly show that the pollen is not evenly distributed over the leaf surface. The pollen tends to accumulate at structures or dents on the leaf surface, including leaf veins and ribs (Figure 4a and 4b). This agrees with previous findings of Pleasants *et al.* [14] who observed the accumulation of pollen in midribs of milkweed plants. However, accumulation was found to be not restricted to midribs but stretched over into adjacent leaf zones. Accumulation can be observed around smaller veins or depression zones of the leaf surface and also in other leaf areas (Figure 3). In addition, pollen was found to adhere to certain structures like trichomes, as in *S. scabiosa* or in *U. dioica* (Figure 4d), leading to relatively higher pollen densities over time compared to the leaves with glossy surface.

Due to the high variation of pollen deposition on the leaves and the relatively small spot size area, an adequate number of images per leaf have to be taken for representative measurements. This question was addressed when we increased the efficiency of the sampling design. Table 1 shows the results of the comparison of different sampling designs. Random sampling, clusters and transects differ in their ability to estimate the mean and the variation of pollen deposition. To optimize the design, we combined transects and clusters in a structured design. This derived design, using a total of 27 images per leaf, describes the pooled data well in both aspects: mean and variation.

The first results of the method show a high variability of pollen deposition within and between leaves. Although this publication does not focus on quantitative measurements of pollen densities on different host plants, some exemplary values observed in the field are depicted in Figures 3 and 4. The observed pollen deposition values from the different plant species in and in close proximity (0–20 m) to the maize fields varied substantially but frequently included maximum pollen deposition values higher than 1,000 to 3,000 pollen/cm^2^ linked to mean values ranging around 100 to 300 pollen/cm^2^. Pollen deposition values of this order of magnitude were found not only on maize, but also on *Rumex, Urtica, Chenopodium, Succisa* and *Centaurea* (Figure 3). Compared to the daily mean values, maximum values of pollen deposition can be found in the range of one to two orders of magnitude higher in accumulation zones. In general, high variation of pollen deposition is in accordance with other studies [15,16,18], but in detail, the variation observed was higher than previously reported.

In the past, some authors considered pollen deposition values over 400 pollen/cm^2^ as ‘very high’ [18]. A risk assessment carried out in the U.S. to assess effects of Bt maize on the Monarch butterfly (*Danaus plexippus*) estimated within-field pollen deposition on host plants between 65 and 425 pollen/cm^2^ [19], but here the method was suitable to assess rather mean values and not the variation. Maximum values reported by studies using ‘leaf prints’ [15–17] ranged from 320 to 972 pollen/cm^2^. Pleasants *et al.* [14] found values up to 1,200 pollen/cm^2^ under exclusion of midrib accumulation zones and on average 1.6 times higher when including midrib zones. However, comparing values is difficult, because the methods and the exposure times differed markedly between the studies. To compare pollen exposure from site to site or year to year the accumulated total pollen deposition over the pollen season is the recommended parameter and basis for standardized measurements [11].

The pollen deposition values observed with digital microscopy are in the same order of magnitude as literature values derived from standardized measurements using the PMF or the Durham trap. Measurements by PMF samplers over six years showed total pollen deposition values up to the range of 2,000 pollen/cm^2^ [20]. In Japan, Kawashima *et al.* [10] recorded a daily maximum value of 1,200 maize pollen/cm^2^ at the field edge and they calculated for the potential total deposition values of up to 15,000 pollen/cm^2^.

Coming to the limitations of the method, the use of digital microscopy on plant leaves is confined to micro-scale measurements e.g., showing up the variation of pollen deposition per leaf, plant and site. Because of the relative small spot area of 5 mm^2^ compared to the total leaf area and the necessary counts of pollen needed for statistical reasons, the effort will strongly increase when mean pollen densities decrease. In practice, we found the method is restricted to areas with higher numbers of pollen deposition e.g., sites within or close to the edge of maize fields. The method is not recommended for meso- or macroscale measurements of spatial or temporal variation (e.g., variation between sites, gradients from the field to greater distances) thus time and effort increases with distance to the field and would become too high compared to other methods. For spatial investigations, e.g., site-to-site comparisons, standardized passive samplers like the PMF are more efficient and therefore recommended. For assessing the temporal variation of pollen shedding and exposure, this can be done in a standardized way at selected sites using active sampling methods like volumetric traps [21]. To estimate the exposure of NTOs with Bt protein via pollen, we suggest to use the method of *in situ* digital microscopy complementary focusing on the evaluation of the micro-scale variation on the leaves, which cannot be achieved by the more standardized technical methods of active and passive sampling.

## 4. Conclusions

The presented method of *in situ* digital microscopy is especially suitable for the analysis of temporal and spatial variation of pollen on receptor surfaces such as plant leaves on a micro scale. The method offers some important advantages over previously used methods to estimate pollen deposition on plant leaves. In this respect, digital microscopy allows *in situ* measurements to be obtained on the spatial and temporal patterns of pollen deposition on host plants. Such data would be much appreciated for the risk assessment of possible non-target effects from Bt maize, in particular lepidopteran larvae, or possibly other herbivorous non-target organisms. Due to the high variation of pollen deposition on a micro scale, direct measurements of pollen deposition on leaf surfaces are not suitable for meso and macro scale evaluations. For environmental risk assessment, which may have regulatory implications, the method is recommended complementary to other methods enabling more standardized measurements of pollen exposure on meso or macro scale (active and passive samplers).

## Figures and Tables

**Table 1:** Results using the different sampling designs to optimize the measurement of the variation of maize pollen deposition on a leaf. Descriptive statistics on the distribution of the maize pollen deposition, values on the base of log-normal distribution. N: number of sample points (images) per leaf [image area: 5 mm^2^].

	**Random Raster**	**Full Transect, Lateral, Mid Leaf**	**5 Transects each with 5 Sample Points**	**6 Clusters each with 5 Sample Points**	**Total**	**Derived Structured Design (Combination of 3 Transects each with 5 Sample Points and 4 Clusters each with 3 Sample Points)**
	**[n/cm^2^]**	**[n/cm^2^]**	**[n/cm^2^]**	**[n/cm^2^]**	**[n/cm^2^]**	**[n/cm^2^]**

N	35	37	25	30	**127**	27
*max*	2787	2302	2745	5448	**5448**	5448
*95% quantile*	1907	1486	2112	2058	**2152**	2289
mean	64	175	72	222	**118**	*130*
*5% quantile*	14	14	14	17	**14**	14
*min*	14	14	14	14	**14**	14
